# Exploring the plant-associated bacterial communities in *Medicago sativa* L

**DOI:** 10.1186/1471-2180-12-78

**Published:** 2012-05-20

**Authors:** Arcangela Frascella, Marco Bazzicalupo, Carla Scotti, Alessio Mengoni

**Affiliations:** 1 Department of Evolutionary Biology, University of Florence, via Romana 17, I-50125, Florence, Italy; 2 Centro di ricerca per le produzioni foraggere e lattiero-casearie (CRA-FLC), viale Piacenza 29, I-26900, Lodi, Italy; 3 Institut de Recherche Interdisciplinaire - IRI CNRS USR3078, Parc de la Haute Borne 50 avenue de Halley, F-59658, Villeneuve d'Ascq Cedex, France; 4 Department of Agricultural Biotechnology, Section of Microbiology, University of Florence, Piazzale delle Cascine 24, 50144, Florence, Italy

## Abstract

**Background:**

Plant-associated bacterial communities caught the attention of several investigators which study the relationships between plants and soil and the potential application of selected bacterial species in crop improvement and protection. *Medicago sativa* L. is a legume crop of high economic importance as forage in temperate areas and one of the most popular model plants for investigations on the symbiosis with nitrogen fixing rhizobia (mainly belonging to the alphaproteobacterial species *Sinorhizobium meliloti*). However, despite its importance, no studies have been carried out looking at the total bacterial community associated with the plant. In this work we explored for the first time the total bacterial community associated with *M. sativa* plants grown in mesocosms conditions, looking at a wide taxonomic spectrum, from the class to the single species (*S. meliloti*) level.

**Results:**

Results, obtained by using Terminal-Restriction Fragment Length Polymorphism (T-RFLP) analysis, quantitative PCR and sequencing of 16 S rRNA gene libraries, showed a high taxonomic diversity as well as a dominance by members of the class *Alphaproteobacteria* in plant tissues. Within *Alphaproteobacteria* the families *Sphingomonadaceae* and *Methylobacteriaceae* were abundant inside plant tissues, while soil *Alphaproteobacteria* were represented by the families of *Hyphomicrobiaceae*, *Methylocystaceae*, *Bradyirhizobiaceae* and *Caulobacteraceae*. At the single species level, we were able to detect the presence of *S. meliloti* populations in aerial tissues, nodules and soil. An analysis of population diversity on nodules and soil showed a relatively low sharing of haplotypes (30-40%) between the two environments and between replicate mesocosms, suggesting drift as main force shaping *S. meliloti* population at least in this system.

**Conclusions:**

In this work we shed some light on the bacterial communities associated with *M. sativa* plants, showing that *Alphaproteobacteria* may constitute an important part of biodiversity in this system, which includes also the well known symbiont *S. meliloti*. Interestingly, this last species was also found in plant aerial part, by applying cultivation-independent protocols, and a genetic diversity analysis suggested that population structure could be strongly influenced by random drift.

## Background

Similar to the intensively studied animal microbioma, plants harbor a wide range of diverse bacteria forming a complex biological community, which includes pathogens, mutualists (symbionts), and commensals [1,2]. Depending on the colonized compartment, these bacteria are rhizospheric (root colonizers), endophytic (colonizing the endosphere, the bulk of internal tissues) and phyllospheric or epiphytic (leaf or stem surface). In recent years plant-associated bacteria (endophytic, epiphytic and rhizospheric) have been widely studied, mainly as promising tools for biotechnological applications [3-7], but investigations have also been carried out on the ecology and taxonomy of plant-associated bacterial communities [8-11]. Despite a high taxonomic diversity, only few bacterial taxa have been found characteristically associated to the majority of plant species, notably members of the *Alphaproteobacteria* class [2,7,8,12,13]. Consequently, the generally accepted idea is that the ability to colonize a plant is not a common, widespread feature present in the soil bacterial community, but preferentially resides in specific taxa which may be considered more ecologically versatile or genetically prone to the association with plants. This last hypothesis has recently been supported by the finding that, at least in the class of *Alphaproteobacteria*, a common gene repertoire seems to be present in all of its plant-associated members [14].

*Medicago sativa* L. (alfalfa) is one of the most important legume crop in temperate areas throughout the world, commonly used as forage or in crop rotation practices to contribute organic nitrogen to the soil via its symbiosis with the nitrogen fixing bacteria [15]*.* Moreover, it is important also for bioenergy production [16] and is one of the most suited plant species for land restoration [17]. Finally, this species, and the diploid relative *M. truncatula* Gaertn. (barrel medic), are among the most studied model species regarding the molecular aspects of plant-bacteria symbiosis, particularly in relation with the alphaproteobacterium *Sinorhizobium* (syn. *Ensifer*) *meliloti*[18-20]. Concerning *S. meliloti*, this species is present in most temperate soils, and, when conditions are suitable, it forms specialized structures, called nodules, in the roots of alfalfa plants where it differentiates into bacteroids [18]. It is assumed that a fraction of bacterial cells is released from dehiscent nodules to soil, giving rise to new free-living rhizobial clones [21]. In the last years *S. meliloti* has been found able to also endophytically colonize the aerial part of other plant species, as rice [22], suggesting the presence of several ecological niches for this species (soil, nodule, other plant tissues).

While the plant-associated bacterial flora of *M. sativa* has never been investigated at the community level, *S. meliloti* population genetics have been extensively studied in the past [23-28], but only on strains isolated from nodules, with a few early studies performed on bacteria directly recovered from soil [29,30], due to the lack of efficient selective culture media. No data have been reported on the presence in natural conditions of *S. meliloti* as endophytes in other plant compartments (such as leaves) and no comparison of soil vs. plant-associated populations has been done.

Based on the above mentioned considerations, there is a need to characterize the bacterial community associated with *M. sativa* in relation to both the potentially important role the class of *Alphaproteobacteria* seems to have as main component of a “core plant-associated bacterial community” in several different plant species [13,31-33], and to the relationships of soil vs. plant-associated populations of the symbiotic alphaproteobacterial partner *S. meliloti*.

In this work we investigated the bacterial communities associated with the legume *M. sativa*, focusing on both the total bacterial community composition and on the presence and populations structure of the symbiotic partner *S. meliloti* in soil and plant tissues*.*

The analysis was conducted by cultivation-independent techniques on alfalfa (*M. sativa*) plants grown in mesocosm pots. The bacterial community associated with *M. sativa* and that of the surrounding soil were analyzed at high (class, family) and low (single species, *S. meliloti*) taxonomic levels by employing Terminal-Restriction Fragment Length Polymorphism (T-RFLP) profiling [33], 16 S rRNA library screening and *S. meliloti*-specific markers [34,35]. These approaches allowed us to explore for the first time the bacterial community composition of such important plant species and the populations of *S. meliloti* without cultivation.

## Results

### Ribotype variability of the bacterial community

The ribotype variability of bacterial communities present in soil and associated to plant tissues (nodules, stems and leaves) was investigated by T-RFLP analysis. A total of 43 samples was analyzed: in particular one pooled soil sample for each one of the three pots, one pooled sample from all the nodules found in each pot and four plants *per* pot (one stem and 2–3 pools of leaves *per* plant). T-RFLP profiles on these samples produced 253 Terminal-Restriction Fragments (T-RFs) or ribotypes after the restriction digestion with two restriction enzymes, *Hinf*I and *Taq*I. 16 S rRNA gene amplification and T-RFLP profiling was also performed on DNA extracted from surface-sterilized seeds, but no bands of 16 S rRNA gene amplification were recovered (data not shown), suggesting a very low bacterial titre in seeds.

**Figure 1:**
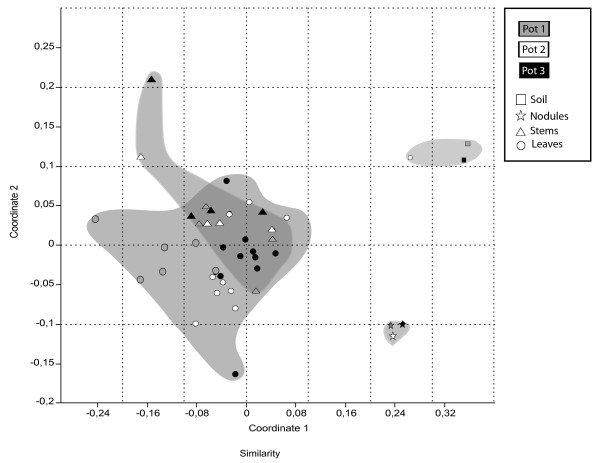
**Pattern of similarities of individual T-RFLP profiles from total community analysis.** The pattern of similarity has been inspected by using Nonmetric Multidimensional scaling (N-MDS) based on Jaccard similarity matrix. Stress of N-MDS = 0.1896. Stars indicate nodules; squares, soils; circles, leaves; triangles, stems. Grey filling, pot 1; white, pot 2; black, pot 3. Samples of the same environment were grey shaded.

Figure 1 shows the pattern of similarity among T-RFLP profiles from total communities as Non-Metric Multidimensional Scaling (N-MDS). Soil and nodule bacterial communities were strongly differentiated from stem and leaf communities, forming relatively tight clusters. Large heterogeneity was detected in leaf and stem communities. To better evaluate the statistical significance of differentiation of communities we employed AMOVA. Most of the variation (71.75%) was due to intra-environment differences (Additional file 1: Table S1). However, significant differences between environments were found (P < 0.0001), in particular between a soil-nodule group and a stem-leaf group.

Interestingly, stem and leaf communities showed a significant (P < 0.0001), though small (pairwise *F*_*ST*_ = 0.05) separation (Additional file 2: Table S2). Moreover, AMOVA on stems and leaves community revealed a statistically significant differentiation between the three pots (P < 0.0001), irrespective of possible grouping (either plant genotype-related or unrelated), suggesting a pot-effect over the taxonomic shaping of the leaf-associated community and no effect of plant genotypes. These data confirmed a previous long-term experiment only addressing *S. meliloti* species [23].

### Taxonomic composition of bacterial communities in soil, nodules and plant aerial parts

T-RFLP analysis has shown that bacterial communities clustered in three groups (soil, nodules and plant aerial parts). In order to elucidate which taxa are mainly represented in the bacterial communities of these assemblies, three 16 S rRNA gene clone libraries were constructed pooling together the DNAs extracted from the samples of each environment; additionally, leaves and stems samples were also pooled together due to their high similarities as mentioned above. Pooled samples did conceivably result in an enrichment of the more shared taxa possibly preventing the detection of taxa associated only to a few individual samples. DNA was used as template to construct three 16 S rRNA libraries; a total of 276 clones (from 78 to 116 *per* library) were sequenced. Sequence analysis revealed, as expected, that the soil community was the most diverse (Shannon H’ = 4.63; Chao1 = 168), while the nodule-associated community was less diverse (Shannon H’ = 1.98; Chao1 = 30), (Additional file 3: Table S3). As a consequence, the library of nodules showed a coverage (85.9%) higher than those of stems + leaves (74.1%) and soil (47.1%).

**Figure 2:**
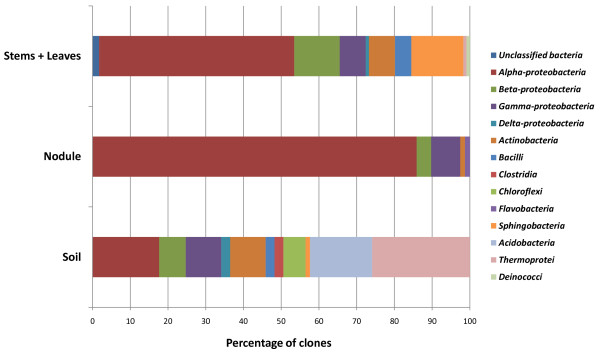
**Representation of bacterial divisions in the 16 S rRNA gene clone libraries.** The percentage of clones accounting for each division with respect to its origin (nodule, stems + leaves, soil) is reported.

The percentages of taxonomic classes detected in the sequences of the clone libraries are reported in Figure 2. Seven classes were represented in both soil and stem + leaf communities, and 4 of them were also found in nodules. *Alphaproteobacteria* were dominant in nodules (as expected, due to the presence of high titres of the symbiotic alphaproteobacterium *S. meliloti*) and in stems + leaves. Also in soil *Alphaproteobacteria* were highly prevalent, but *Acidobacteria* and *Crenarchaeota* were also abundant. *Flavobacteria* were found only in nodules, however a low presence in the other environments cannot be excluded, especially in relation to the lower coverage of the respective libraries. *Beta-* and *Gammaproteobacteria* and *Actinobacteria* were found in all three libraries.

**Figure 3:**
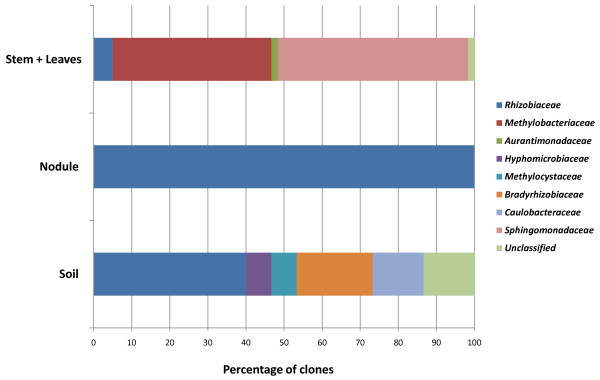
**Distribution of the recovered families in*Alphaproteobacteria*with respect to their origin (nodule, stems + leaves, soil).** The percentage of clones present in the libraries for each family is reported.

Concerning *Alphaproteobacteria*, only members of the *Rhizobiaceae* family were found in nodules, with all sequences assigned, as expected, to the *Sinorhizobium/Ensifer* genus (Figure 3). *Alphaproteobacteria* present in soil belonged to the *Rhizobiaceae*, *Bradyrhizobiaceae*, *Methylocystaceae, Hypomicrobiaceae* and *Caulobacteraceae* families*. Rhizobiaceae, Aurantimonadaceae* and *Methylobacteriaceae*, all belonging to the *Rhizobiales*, plus taxa of the order *Sphingomonadales*, were found in the stem + leaf library. The absence of sequences assigned to the *Sinorhizobium/Ensifer* genus from stem + leaves and soil libraries, though this species was found by qPCR in both these environments (see the following paragraph), could be due to its low abundance and to the relatively low coverage of clone libraries.

### Detection and diversity of *sinorhizobium meliloti* in soil and plant tissues

Aiming to analyze presence and diversity of *S. meliloti,* we firstly estimated the population size by qPCR, using two species-specific primer pairs which amplify chromosomal (*rpo*E1) and megaplasmidic loci (*nod*C on pSymA), respectively [35]. The obtained results are reported in Table 1. Relatively higher titers of *S. meliloti* DNA were detected in root nodules, while lower values were obtained in soils, leaves and stems. Interestingly, nodule titers of *S. meliloti* DNA detected by *rpoE* marker were higher than those estimated by *nodC* marker (roughly one order of magnitude). The viable titers of *S. meliloti* cells from crushed nodules of *M. sativa* plants usually ranged from 2.1x10^8^ to 5.0x10^8^cells/g of fresh tissue (data not shown), suggesting that the titers from *nodC* marker are a better proxy of the number of bacteria involved in the symbiotic nitrogen-fixing process.

**Table 1:** **Titers of*S. meliloti*in soil and plant tissues§**

***Sample***	***Titers***	
	***rpoE1*-based**	***nodC*-based**
**Pot 1**		
Soil	4.92 ± 2.82 x 10^4^	2.78 ± 0.63 x 10^4^
Nodules	3.07 ± 0.67 x 10^9^	4.25 ±1.24 x 10^8^ **
Stems	2.73 ± 1.21 x 10^4^	3.22 ±2.4 x 10^3^ *
Leaves	8.65 ± 4.04 x 10^3^	4.28 ± 1.23 x 10^3^
**Pot 2**		
Soil	1.16 ± 0.33 x 10^4^	2.88 ± 1.09 x 10^4^
Nodules	1.20 ± 0.50 x 10^10^	1.01 ± 0.10 x 10^9^ **
Stems	2.37 ± 0.49 x 10^3^	1.13 ± 0.15 x 10^3^
Leaves	9.74 ± 5.08 x 10^2^	2.34 ±0.78 x 10^2^
**Pot 3**		
Soil	2.70 ± 0.41 x 10^5^	7.42 ±0.93 x 10^4^ *
Nodules	6.02 ± 1.45 x 10^9^	2.02 ± 3.22 x 10^7^ **
Stems	4.91 ± 0.95 x 10^5^	1.07 ± 3.74 x 10^5^
Leaves	5.54 ± 2.83 x 10^3^	5.21 ± 3.01 x 10^3^

§Titers were estimated by qPCR [35] with *rpoE1* and *nodC* markers and are expressed as n. of gene copies/g of tissue or soil; ± standard deviation from triplicate experiments. Asterisks indicate significant differences between estimates based on *rpoE1* and *nodC* markers (*, P < 0.05; **, P < 0.01).

**Figure 4:**
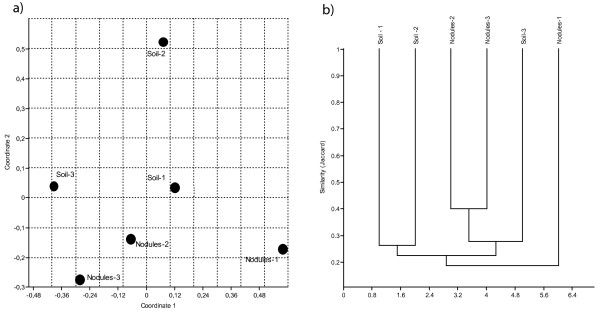
**a) Non-metric MDS plot of similarities of IGS-T-RFLP profiles from*S. meliloti*population analysis.a)** The pattern of similarity of *S. meliloti* populations has been inspected by using Non-metric Multidimensional scaling (N-MDS) based on Jaccard similarity matrix. Stress = 0.0898. **b)** Cluster analysis based on Jaccard similarity matrix. Scale bar represents Jaccard similarity coefficient

Then, to inspect the genetic diversity of *S. meliloti* populations present in the different environments, the amplification of the 1.3 kbp long 16 S-23 S ribosomal intergenic spacer (IGS) which proved to be an efficient marker for the study of *S. meliloti* populations [34], was attempted. Only DNAs from nodules and soil gave a PCR product, probably as a result of the low bacterial titers and high content in inhibitors present in DNA extracted from stems and leaves. Consequently, nodule tissue was taken as representative of the plant environment and was compared with soil. A total of 121 different IGS-T-RFs (16 S-23 S ribosomal intergenic spacer Terminal-Restriction Fragments) was detected after digestion with four restriction enzymes (*Alu*I, *Msp*I, *Hinf*I, *Hha*I) in the six DNA samples (three from soil, three from nodules), after IGS amplification and T-RFLP profiling (Additional file 4: Figure S1a). Most of the 121 detected IGS-T-RFs (71.9%) were detected in one sample out of 6, while 8 (6.6%) IGS-T-RFs were present in all six samples (Additional file 4: Figure S1b). Moreover, from 25.5 to 53.3% of IGS-T-RFs present in soil were also detected in nodules and from 31.4 to 40.1% of IGS-T-RFs present in nodules were detected in the respective soil sample. Figure 4 shows the similarity relationships between IGS-T-RFLP profiles. Non-metric MDS plot of IGS-T-RFLP profiles (Figure 4a) showed a possible separation of nodule and soil populations on the second dimension. In particular, the nodule population in pot 1 was more separated from the soil population of the same pot and from the populations of the other pots. On the contrary, nodule populations of pots 2 and 3 were the closest ones, with soil population of pot 3 in the same cluster (Figure 4b), suggesting a possible effect of plant genotype as previously shown [23,36]. However, in agreement with the high number of single-sample haplotypes detected, an AMOVA carried out to evaluate the variance contribution to a hypothetical differentiation of soil and nodule *S. meliloti* population showed that 17.37% only of variance was attributed to a soil-nodule separation, the remaining 82.63% of variance being due to among-nodules and among-soil differences. Additionally, no statistical significant separation (P < 0.46) was detected for groupings based on the two plant genotypes present in the mesocosms.

## Discussion

In recent years there has been an increasing interest in exploring the bacterial flora associated with plants [37-41]. A recent field survey indicates [8] that plant aerial parts (leaves) harbor complex, but highly variable, bacterial communities, and that only a small number of bacterial taxa (mainly belonging to *Alphaproteobacteria*) are plant-specific. In the experiments reported here, as in the majority of the reports on endophytic microflora, we refer to endophytic and epiphytic bacteria indicating all those that are inside the plant tissue or strongly adhering to the plant surface, such as they resist washing and sterilization (or their DNA is retained by plant tissue), therefore a more correct definition could be “plant-associated bacteria”.

The present study shows that root nodules and aerial parts of *Medicago sativa* plants grown in mesocosm conditions, harbor distinct bacterial communities with specific signatures at the class, family and species levels and that these communities do not mirror soil bacterial communities.

Initially, T-RFLP profiles allowed us to show that bacterial communities present in the different environments (soil, nodules, stems and leaves) were strongly differentiated and in particular that a large heterogeneity was present between leaves of individual plants, though soil profiles were highly similar. Moreover a clear separation between above-ground (stem and leaves) and below-ground environments (soil and nodules) was detected. An analysis of the clone libraries, prepared from above-ground and below-ground pooled samples, revealed an uneven distribution of bacterial classes, with a marked pattern highlighting the class of *Alphaproteobacteria* as the more abundant in plant tissues (this class represented half of the clones in the stem + leaf library). The same uneven pattern was then observed, at lower taxonomic ranks, within the *Alphaproteobacteria*, with sequences of clones belonging to members of the *Methylobacteriaceae* and *Sphingomonadaceae* families being more abundant in stem than in soil and nodules. *Methylobacteria* and *Sphingomonadaceae* have been found as endophytes in a number of plants [8,12,31,33,42-45] and it is believed that this group of bacteria may take advantage from living as plant-associated, thanks to its ability to utilize the one-carbon alcohol methanol discharged by wall-associated pectin metabolism of growing plant cells.

Concerning root nodule bacterial communities, obtained data indicated that very diverse bacterial taxa are associated with nodules, the most represented being the specific rhizobial host of *M. sativa*, the alphaproteobacterium *S. meliloti*. However, additional taxa have been found, including members of *ActinobacteriaFlavobacteriaGammaproteobacteria* and *Betaproteobacteria*, which may have some additional plant growth-promoting activities (see for instance [46,47]).

In soil, *Acidobacteria* was one of the most important divisions (in terms of number of clones in the library) and was present exclusively in the soil clone library, in agreement with many previous observations [48,49]. A relatively high presence of *Archaea* (*Thermoprotei*) was also found. Checking the 16 S rRNA gene sequences present in the Ribosomal Database for 799f/pHr primer annealing, we found that PCR amplification from *Thermoprotei* was theoretically possible with this primer pair (data not shown). The presence of *Archaea* in the soil is not unexpected [50] and could be linked also to the anoxic or nearly anoxic conditions present in the bottom of the pot. However, since the low coverage of soil clone library, the presence of many other additional taxa, as well of different proportions of those found here cannot be excluded. In addition, it should be mentioned that differences between soil and plant-tissues bacterial communities could also be ascribed to the different DNA extraction protocols we were obliged to use, since a unique protocol (bead-beading protocol for both soil DNA and plant DNA) failed in a successful extraction of DNA from both soil and plant tissues (data not shown). A similar technical need was encountered by other authors also [33], which renders the study of the relationships between plant-associated and soil bacterial communities still at its beginning.

At the lowest taxonomic rank here investigated, within the species *S. meliloti*, we detected the presence of this species in all environment analyzed (soil, nodules and plant aerial tissues). This finding is confirming earlier reports on the ability of *S. meliloti* to behave as an endophytic strain, colonizing all plant compartments, besides being a root symbiont of legumes [22], and suggest a potential higher genetic variability of *S. meliloti* population, and, from the other side, potential new ecological and functional roles for this species, not investigated so far[29,51,52]. Unfortunately, the low population size of *S. meliloti* in stems and leaves and the possible presence of PCR inhibitors (plant DNA or phenolic compounds, for instance) did not permit the amplification of 16 S-23 S rRNA intergenic region from plant aerial parts to obtain information about the genetic diversity and structure of *S. meliloti* population resident in plant aerial part. No hypothesis could then be drawn about the relationships between this population and those of soil and nodules. Concerning *S. meliloti* populations present in soil and nodules, similar values for diversity were detected in nodules and in soil, suggesting that both environments harbor a consistent fraction of the population’s genetic diversity. Interestingly, most of the T-RFs were detected in one sample only, and a very small fraction of T-RFs was shared among all samples, though the original soil material was homogeneous and should, in theory, contain the same *S. meliloti* haplotypes. Therefore, *S. meliloti* populations from all the three mesocosms investigated were highly differentiated between each other and, as expected from previous studies on *S. meliloti*[23] and on *Bradyrhizobium*[53], no statistically significant plant genotype- related haplotypes were detected.

A possible explanation of such findings could be linked to the relatively low titers of *S. meliloti* in soil (10^4^-10^5^ cells/g), which is roughly 1/10,000 of the total bacterial community of soil (estimated at ~10^9^ 16 S rRNA gene copies/g of soil by qPCR, data not shown). Such estimated *S. meliloti* titers were similar to those previously observed in other soil and plant tissues [35] and in line with those normally found in soil with viable (Most Probable Number, MPN) estimates [26,54]. As a consequence of this low population size, founder effect and genetic drift are likely to be among the main shaping forces of *S. meliloti* population in this experimental set-up, perhaps permitting the fixation of sample-specific haplotypes by simple chance [55]. Regarding the nodule-soil relationships, though our experiments did not directly address this issue, the reported *S. meliloti* population analysis suggests the presence of somewhat nonoverlapping soil and nodule population fractions, even if no specific patterns of soil and nodule populations were detected. The presence of different rhizobial haplotypes in nodules and soil was previously found in chickpea [51] and clover [52], though no simple conclusion could be drawn, because of limited sampling. However, as for total bacterial community analysis, it should be mentioned that the use of two different DNA extraction protocols for soil and plant DNA may have produced some bias in the proportion of the different haplotyes detected.

## Conclusion

In conclusion, we show on *M. sativa* that its associated microflora, though highly variable, is mainly related to the presence of *Alphaproteobacteria*. This class has an uneven presence of families in stems + leaves, nodules and soil. We then speculated that a sort of “pan-plant-associated bacterial community” may be composed of a large plethora of “accessory” taxa, which are occasionally associated with plants, and a small number of “core” taxa (e.g. *Alphaproteobacteria* families) which, on the contrary, are consistently found in the plants. Moreover, within *Alphaproteobacteria* the specific alfalfa symbiotic species *S. meliloti*, abundant as symbiont in root nodules, was also detected in soil and in leaves, with potentially different populations, suggesting a more complex interplay of colonization of multiple environments (soil, root nodules, other plant tissues) by this species.

## Methods

### Experimental design and sampling procedure

A controlled experiment was set-up in mesocosms composed of three pots (numbered 1, 2, 3) containing *Medicago sativa* (alfalfa) plants grown at CRA-FLC Lodi, Italy, in outdoor conditions. Two of the three pots were planted with the same line of alfalfa (1x5) while the third pot was planted with a different line (5x7). The pots (cylinders of 25 cm diameter x 80 cm depth) with a drainage layer on the bottom, were filled with a sandy loam non-calcareous soil (57.8% sand, 32% silt, 10.2% clay, 1.7% organic matter and 0.09% total N; pH 6.7) in which alfalfa has never been grown. Phosphorus and potassium equivalent to 120 Kg ha^-1^ of P_2_O_5_ and 180 Kg ha^-1^ of K_2_O were distributed into the soil, while no mineral N was added; irrigation was not limiting. Twenty plants/pot (density equivalent to 400 plants m^-2^) were transplanted in March 2008 and allowed to grow until the 2^nd^ year (the end of September 2009), when plant aerial parts of 12 plants were harvested and the pots were opened to allow sampling of the whole eye-detectable nodules present (approximately 80–100 of various sizes per pot) and of bulk soil. Roots were excluded from the analysis since the presence of small nodules or nodule primordia could not be excluded, possibly inducing a strong bias in the estimation of “non-nodule-associated root colonizers”. The plant sample size was chosen on the basis of a previous analysis of plant-by-plant variation in which the overall diversity of communities did not change from 2 to 30 plants (unpublished data and [8]). Stems, leaves (pools of around 10 leaves per plant) and nodules were washed with water and with 10 mM MgSO_4_ twice to remove most soil and dust particles and eliminate bacteria loosely adhering to the surface and then surface sterilized with 1% HClO for 1 min. Samples of soil, nodules, stem and leaves were then stored at −80°C from 1–2 weeks before DNA extraction.

A control of seed-borne bacteria was also prepared with seeds of *M. sativa* surface sterilized with 1% HgCl_2_.

*S. meliloti* viable titres in sterilized nodules have been estimated by serial dilution of crushed nodules as previously reported [54].

### DNA extraction real-time PCR and T-RFLP profiling

DNA was extracted from soil by using a commercial kit (Fast DNA Spin kit for soil, QBiogene, Cambridge, UK) following the manufacturer’s instructions. DNA extraction from plant tissues and surface sterilized control seeds was performed by a 2X CTAB protocol as previously described [56]. The 16 S rRNA gene pool of total bacterial community was amplified from the extracted DNA with primer pairs 799f (labeled with HEX) and pHr which allow the amplification of most bacterial groups without targeting chloroplast DNA [33]. PCR conditions and Terminal-Restriction Fragment Length Polymorphism (T-RFLP) profiling were as previously reported [8], by using *Hinf*I and *Taq*I restriction enzymes. For sinorhizobial populations, T-RFLP was carried out on 16 S-23 S ribosomal intergenic spacer amplified from total DNA (IGS-T-RFLP) with *S. meliloti* specific primers and *AluI* and *HhaII* restriction enzymes, as already reported [34]. Real-Time PCR (qPCR) for quantification of *S. meliloti* DNA was carried out on *rpo*E1 and *nod*C loci, as previously reported [35]; two different calibration curves were constructed, one for soil samples and the other one for plant samples, by using as template DNA extracted from sterile soil (without presence of *S. meliloti*) and from sterile plant (grown in petri dishes), both spiked with serial dilutions of known titres of *S. meliloti* cells, as previously reported [35]. Controls with *S. medicae* WSM419 DNA were included in both IGS-T-RFLP and qPCR, for *S. meliloti* species-specificity check [35].

### Library construction and sequencing

Amplified (with 799f and pHr primer pair) 16 S rRNA genes from DNA extracted from soil, nodules, pooled stems and leaves of a 1:1:1 mix of all pots were inserted into a pGemT vector (Promega, Fitchburg, WI, USA) and cloned in *E. coli* JM109 cells. Positive clones were initially screened by white/blue coloring and the inserted amplified 16SrRNA genes sequenced. Plasmid purification and sequencing reactions were performed by Macrogen Europe Inc. (Amsterdam, The Netherlands). The nucleotide sequences obtained were deposited in Gen- Bank/DDBJ/EMBL databases under accession numbers from HQ834968 to HQ835246.

### Data processing and statistical analyses

For qPCR data, 1-way ANOVA with Tukey post hoc test was employed. Analyse-it 2.0 software (Analyse-It, Ldt., Leeds, UK) was used for both tests. For T-RFLP, chromatogram files from automated sequencer sizing were imported into GeneMarker ver. 1.71 software (SoftGenetics LLC, State College, PA, USA) by filtering with the default options of the module for AFLP analysis. Peaks above 100 fluorescence units and whose size ranged from 35 to 500 nt were considered for profile analysis. Only the presence/absence of peaks was considered as informative data from the chromatograms. Statistical analyses were performed on a binary matrix obtained as previously reported [8]. Past 2.02 [57] software package was used to compute Non-Metric Multidimensional Scaling (N-MDS). To test the distribution of the variance of T-RFLP profiles within plant tissues and among pots, Analysis of Molecular Variance (AMOVA [58]) was applied using Arlequin 3.5.1.2 software (http://cmpg.unibe.ch/software/arlequin3/). Although developed for population genetic analysis, the general procedure implemented by AMOVA is flexible enough to estimate the statistical significance of groups of bacterial communities as reported previously [13,42,59]. Pairwise F_*ST*_ distances [60] between T-RFLP profiles of plant tissues and soils were used to infer a Neighbor-Joining dendrogram with the MEGA4 software [61].

Partial 16 S rRNA sequences were manually inspected for quality, then aligned and clustered with the furthest neighboring algorithm with the module present in Mothur v.1.12.3 [62]. Diversity indices (Shannon H’ and Chao-1) were calculated with the same software. Library coverage was estimated with the formula *C = 1-(n/N)*[63], where *n* is the number of singletons (defined at 97% sequence identity in Mothur) that are encountered only once in the library and *N* is the total number of sequenced clones. Taxonomic assignment was performed with the Classifier module present in Ribosomal Database Project 10 website [64] (http://rdp.cme.msu.edu/) at 80% confidence threshold. Sequences with 97% similarity were treated as a single Operational Taxonomic Unit (OTU). Sequences (one for each OTU) were aligned with the 16 S rRNA gene sequences of the closest match retrieved from NCBI databases, using MUSCLE [65] and a Neighbor-Joining dendrogram was constructed using MEGA4 [61]. Phylogenetic inference and evolutionary distance calculations were generated using the Maximum Composite Likelihood; 1000 bootstrap replicates were used to obtain confidence estimates for the phylogenetic trees.

## Competing interests

The authors declare that they have no competing interests.

## Authors’ contributions

FP performed most of the analyses, prepared the figures and contributed in writing the draft of the manuscript. This work is part of FP PhD thesis. AF and LS gave technical assistance to the experimental work during the preparation of their master degree theses. CS settled the mesocosm experiment and assisted in the samplings. EGB, MB, FP and AM conceived the idea and contributed in performing part of the analyses and in drafting the manuscript. All authors have given final approval of the version to be published.

## Supplementary Material

Additional file 1 **Table S1.** Hierarchical analysis of differentiation between bacterial communities. AMOVA was performed with T-RFLP profiles from samples of the four different environments (soil, nodules, stems and leaves). Data show the degrees of freedom (d.f.), the sum of squared deviation, the variance component estimate, the percentage of total variance contributed by each component, and the probability (*P*) of obtaining a more extreme component estimate by chance alone, estimated from 10,000 permutations. Click here for file

Additional file 2 **Table S2.** Matrix of pairwise FST values. Statistical significance (p < 0.05) has been computed after 1000 random permutation; n.s., not significant. Only below diagonal values are reported. Click here for file

Additional file 3 **Table S3.** Statistical analysis of 16SrRNA gene clone libraries. OTUs were arbitrarily defined at 97% sequence identity based on Mothur clustering. Confidence intervals at 95% are given in parentheses. Coverage is defined C = [1 − (n/N)] × 100, where n is the number of unique clones, and N is the total number of clones examined. Click here for file

Additional file 4 **Figure S1.*** S. meliloti* IGS-T-RFLP profiling of nodule and soil samples. A), the schematic representation of the binary matrix of IGS-T-RF presence (black) and absence (empty cell); the IGS-T-RF number is reported on the right side of each row. B) The occurrence of “private” and “public” IGS-T-RFs. The percentage of total number of scored IGS-T-RFs is reported for T-RFs present from 1 to all 6 samples analyzed. Click here for file
