# Supplementary material for: Exploring the plant-associated bacterial communities in Medicago sativa L
Source: BMC Microbiol. 2012 May 20;12:78. doi: 10.1186/1471-2180-12-78 (PMC3412730; doi:10.1186/1471-2180-12-78)
Supplement: Additional file 1 — Table S1. Hierarchical analysis of differentiation between bacterial communities. AMOVA was performed with T-RFLP profiles from samples of the four different environments (soil, nodules, stems and leaves). Data show the degrees of freedom (d.f.), the sum of squared deviation, the variance component estimate, the percentage of total variance contributed by each component, and the probability (P) of obtaining a more extreme component estimate by chance alone, estimated from 10,000 permutations. [file 1471-2180-12-78-S1.doc]

**Table S1**. Hierarchical analysis of differentiation between bacterial communities.*

| ***Source of variation*** | ***d.f.*** | ***Sum of squares*** | ***Variance components*** | ***Percentage of variation*** | ***P-value*** |
| --- | --- | --- | --- | --- | --- |
| **Among environments** | 3 | 198.346 | 6.13749 | 28.25 | <0.0001 |
| **Within environments** | 39 | 607.933 | 15.58803 | 71.75 |  |
| **Total** | 42 | 806.279 | 21.72552 |  |  |

* AMOVA was performed with T-RFLP profiles from samples of the four different environments (soil, nodules, stems and leaves). Data show the degrees of freedom (d.f.), the sum of squared deviation, the variance component estimate, the percentage of total variance contributed by each component, and the probability (*P*) of obtaining a more extreme component estimate by chance alone, estimated from 10,000 permutations.
