# Supplementary material for: Exploring the plant-associated bacterial communities in Medicago sativa L
Source: BMC Microbiol. 2012 May 20;12:78. doi: 10.1186/1471-2180-12-78 (PMC3412730; doi:10.1186/1471-2180-12-78)
Supplement: Additional file 2 — Table S2. Matrix of pairwise FST values. Statistical significance (p < 0.05) has been computed after 1000 random permutation; n.s., not significant. Only below diagonal values are reported. [file 1471-2180-12-78-S2.doc]

**Table S2**. Matrix of pairwise *F_ST_* values. Statistical significance (p<0.05) has been computed after 1000 random permutation; n.s., not significant. Only below diagonal values are reported.

|  | **Soil** | **Leaves** | **Stem** | **Nodules** |
| --- | --- | --- | --- | --- |
| **Soil** | 0 |  |  |  |
| **Leaves** | 0.42329 | 0 |  |  |
| **Stem** | 0.49207 | 0.05492 | 0 |  |
| **Nodules** | 0.46885 n.s. | 0.43966 | 0.50114 | 0 |
