# Supplementary material for: Exploring the plant-associated bacterial communities in Medicago sativa L
Source: BMC Microbiol. 2012 May 20;12:78. doi: 10.1186/1471-2180-12-78 (PMC3412730; doi:10.1186/1471-2180-12-78)
Supplement: Additional file 3 — Table S3. Statistical analysis of 16SrRNA gene clone libraries. OTUs were arbitrarily defined at 97% sequence identity based on Mothur clustering. Confidence intervals at 95% are given in parentheses. Coverage is defined C = [1 − (n/N)] × 100, where n is the number of unique clones, and N is the total number of clones examined. [file 1471-2180-12-78-S3.doc]

**Table S3**. Statistical analysis of 16S rRNA gene clone libraries.

|  | **Statistics** | | | **Diversity indices** | |
| --- | --- | --- | --- | --- | --- |
|  | N. of sequences | N. of OTUs* | Library coverage (%)** | Chao1 | Shannon (H’) (Diversity) |
| **Soil** | 85 | 58 | 47.1 | 168 (105-311) | 4.63 |
| **Stems+leaves** | 116 | 46 | 74.1 | 100 (67-187) | 3.62 |
| **Nodules** | 78 | 16 | 85.9 | 30 (19-72) | 1.98 |

*OTUs were arbitrarily defined at 97% sequence identity based on Mothur clustering. Confidence intervals at 95% are given in parentheses.

** Coverage is defined C = [1 − (n/N)] × 100, where n is the number of unique clones, and N is the total number of clones examined
