# Supplementary material for: Exploring the plant-associated bacterial communities in Medicago sativa L
Source: BMC Microbiol. 2012 May 20;12:78. doi: 10.1186/1471-2180-12-78 (PMC3412730; doi:10.1186/1471-2180-12-78)
Supplement: Additional file 4 — Figure S1. S. meliloti IGS-T-RFLP profiling of nodule and soil samples. A), the schematic representation of the binary matrix of IGS-T-RF presence (black) and absence (empty cell); the IGS-T-RF number is reported on the right side of each row. B) The occurrence of “private” and “public” IGS-T-RFs. The percentage of total number of scored IGS-T-RFs is reported for T-RFs present from 1 to all 6 samples analyzed. [file 1471-2180-12-78-S4.pdf]

a

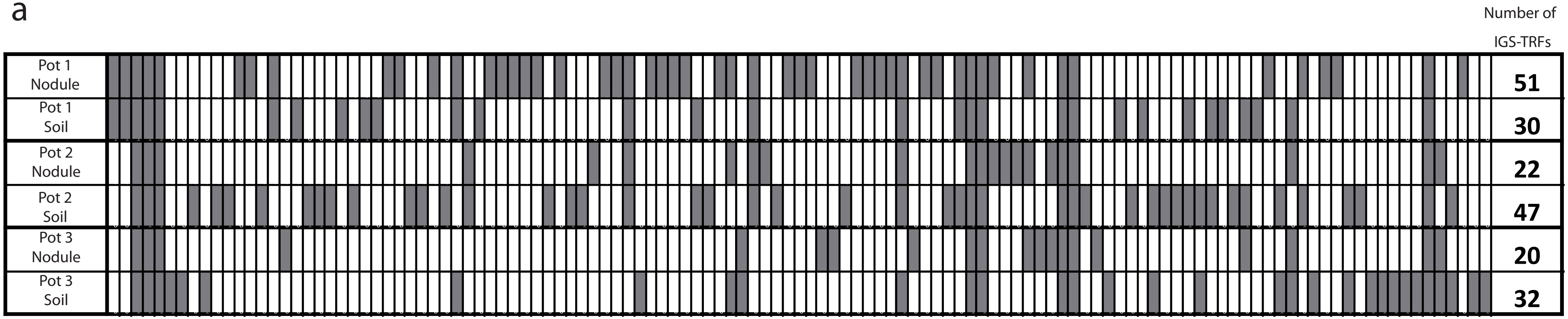

Number of IGS-TRFs
Pot 1 Nodule
Pot 1 Soil
Pot 2 Nodule
Pot 2 Soil
Pot 3 Nodule
Pot 3 Soil
51
30
22
47
20
32

b

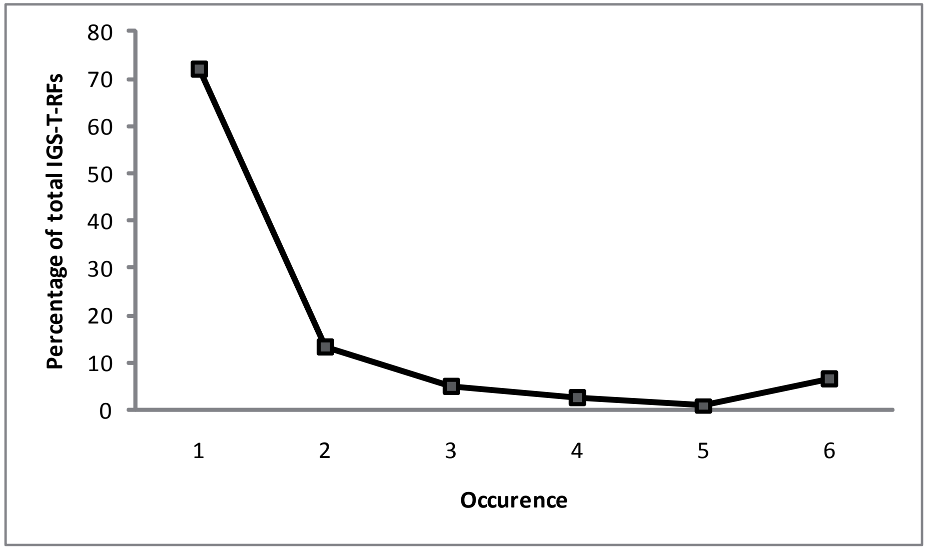

Percentage of total IGS-T-RFs
80
70
60
50
40
30
20
10
0
1
2
3
4
5
6
Occurence
